# Anodal tDCS Over the Right Temporoparietal Junction Lowers Overbidding in Contests

**DOI:** 10.3389/fnins.2019.00528

**Published:** 2019-06-11

**Authors:** Minda Wang, Jianbiao Li, Dahui Li, Chengkang Zhu

**Affiliations:** ^1^School of Economics and Management, Southeast University, Nanjing, China; ^2^Reinhard Selten Laboratory, China Academy of Corporate Governance, Nankai University, Tianjin, China; ^3^School of Economics, Shandong University, Jinan, China; ^4^Department of Economics and Management, Binhai College, Nankai University, Tianjin, China; ^5^Labovitz School of Business & Economics, University of Minnesota Duluth, Duluth, MN, United States

**Keywords:** overbidding, rTPJ, contest, tDCS, nash equilibrium

## Abstract

Overbidding, which means bidding over the Nash equilibrium, is commonly observed in competitive social interactions, such as a contest or auction. Recent neuroscience studies show that the right temporoparietal junction (rTPJ) is related to overbidding and associated with inferring the intentions of others during competitive interactions. The present study investigates the neural underpinnings of overbidding and how the rTPJ impacts bidding behavior by using tDCS to modulate the activation of the rTPJ. Participants completed a two-person proportional prize contest, in which overbidding was frequently observed and each participant's share of the prize was equal to the individual's expenditure divided by the aggregated expenditure. We observed a significant tDCS effect, i.e., participants' average expenditure and overbidding rate were significantly reduced in the anodal stimulation compared with the cathodal and sham stimulation. Possible explanations include that enhanced activity in the rTPJ via the anodal stimulation increased the accuracy of a participant's inference of the strategies of others, or a participant's concern for others, and thus helped the participant bid optimally. Our findings provide evidence supporting that the activation of the rTPJ in contests affects overbidding and bidding strategy, and further confirm that the rTPJ is involved in the inference of mental states in a competition context.

## Introduction

When multiple agents participate in a completion for a single prize or part of a prize, their final expected payoffs are generally the expected prizes won from the competition minus the cost of their effort. The expected prizes are equal to the probability of winning the prize or their probable shares of the prize times the prize value. However, the probability of the winning prize or the share of the prize is positively correlated with the cost of their effort. The more the cost is, the higher the probability of winning is. Therefore, a participant must trade-off between the expected payoff and the cost of effort, based on their belief about what their opponents may choose. The solution of this trade-off, with the assumption of individual rationality, leads to the optimal bid function which maximizes a participant's expected payoff given others' choices. This optimal choice is termed as Nash equilibrium in the economics and game theory literatures. A replicated observation in controlled lab experiments is that participants bid higher than the Nash equilibrium on average and that the total expenditure by all participants exceeds the value of the prize in some cases (Sheremeta, 2013; Dechenaux et al., 2015). This phenomenon, i.e., participants tend to exert more effort which exceeds the Nash equilibrium in a competition is termed as overbidding (Sheremeta, 2013; Chowdhury et al., 2014; Dechenaux et al., 2015; Mago et al., 2016). A similar phenomenon also frequently occurs in common value auctions, known as the winner's curse (Thaler, 1988). A comprehensive survey shows that 28 behavioral studies observed overbidding; overbidding rate ranged from 10 to 256%; and the median overbidding rate was 72% (Sheremeta, 2013). In addition, overbidding may cause significant social welfare waste (Tullock, 1980) and inhibit innovation and economic growth (Abbink and Serra, 2015).

Overbidding often occurs in costly competitions that resemble a contest. In a contest, agents need to invest part of their endowment to compete for a single prize or part of a prize, while their total earnings equal to the remainder endowment plus the prize they win. In the contest, costly effort is irreversible, which means that the effort exerted is unrefundable but determines the outcome of winning or losing. Meanwhile, a player's expenditure may affect the probability of winning the prize or their share of the prize, which induces uncertainty about the results. Contests are very common in the human society, such as R&D race between firms, patent competition, arm conflict, rent seeking, and education investment.

In a contest, a participant's expenditure may increase the probability of winning the prize or the share of the prize. However, the expenditures will exhaust irreversible resource. Participants are thus motivated to compute the Nash bidding strategy, i.e., to maximize the expected payoff based on the belief about what their opponents are likely to do. Nash equilibrium strategy is, to some extent, a normative choice with which most players have an intrinsic desire to comply with (Fehr and Schurtenberger, 2018). To find out the Nash equilibrium strategy, participants need to infer what the majority of individuals will do and assume their opponents will choose this strategy. If a participant cannot predict their opponents' strategies correctly, the participant may choose a suboptimal strategy, which may lead to overbidding. For instance, behavioral economics studies found that overbidding occurred when participants held the belief that opponents would invest higher than Nash equilibrium (Rockenbach and Waligora, 2016) and apply non-best response function (Price and Sheremeta, 2011; Chowdhury et al., 2014; Rockenbach and Waligora, 2016; Sheremeta, 2016). Another behavioral study has shown that subjects of higher strategic reasoning level might spend more closely to Nash equilibrium (Lim et al., 2014). Therefore, mental state inference and mentalizing related strategy computation (Hampton et al., 2008) could be the neural decision making process underlying overbidding and suboptimal bidding behavior. In addition, if a participant detects any deviation between their own estimate and the actual strategy that the opponent applies at the beginning of the game, they may try to adapt their own strategic choices through allocating more attention, resources, and effort to infer the opponent's strategy in the next competitive interactions (Bitsch et al., 2018). Therefore, the neural function of belief adjustment is highly relevant to overbidding.

One neuroimaging study has revealed that activity in the right temporoparietal junction (rTPJ) was associated with overbidding (Van den Bos et al., 2013). Another study has also found that participants' bids were greater than the optimal in the initial round and that rTPJ was activated when some participants won the auction (Van den Bos et al., 2013). rTPJ was a key brain region which is involved in social cognition such as self and other reflection (Decety and Lamm, 2007; Murray et al., 2012). A long line of neuro-imaging studies have shown that activation of rTPJ, one of the most mentioned regions in ToM neural networks (Rilling et al., 2004; Decety and Lamm, 2007), was a crucial part of the neural network underlying decision making in competition (Assaf et al., 2009; Halko et al., 2009). Many fMRI studies have indicated that rTPJ was commonly related to mental state inference (Assaf et al., 2009; Halko et al., 2009; Votinov et al., 2015; Sugimoto et al., 2016; Tsoi et al., 2016) or belief adaption (Hampton et al., 2008; Bitsch et al., 2018) in competitive situations. rTPJ was also found to be very important to collaborative social interactions during economic exchanges (Bilek et al., 2015; Tang et al., 2016). Furthermore, numerous studies have confirmed that rTPJ was involved in the strategic computation process when participants compete against their human opponents (Coricelli and Nagel, 2009; Carter et al., 2012), especially when more sophisticated participants needed to anticipate the opponent's mental state (Bhatt et al., 2010; Volz et al., 2015) or estimate the influence of their actions on the opponents' strategy choice (Hertz et al., 2017; Hill et al., 2017). Taking these together, it is reasonable to assume that rTPJ is crucial in contests and may impact bidding behavior and overbidding through mental state inference and strategic computation.

While fMRI studies are able to provide correlational diagnoses, transcranial direct current stimulation (tDCS) can establish a more direct link between a region of interest and a particular function (Filmer et al., 2014; Sellaro et al., 2016; Li et al., 2017). Prior tDCS studies (Gan et al., 2013; Sellaro et al., 2015) have found that excited neural activities of rTPJ enhanced the capacity of integrating intention and outcomes information in moral judgment experiments. Santiesteban et al. (2012, 2015) have confirmed that anodal stimulation of rTPJ enhanced the ability to take the other's visual perspective. Recently, researchers have found that strengthening rTPJ decreased deception in moral hypocrisy (Tang et al., 2017), Mai et al. (2016) has also observed that cathodal tDCS on rTPJ made people less capable of inferring others' intentions and emotions. However, there has not yet been a tDCS study which examines whether a change of activity in rTPJ influences overbidding.

The goal of the current research is to clarify the casual involvement of the rTPJ in overbidding in contest by using tDCS technique to modulate the activity in rTPJ. We compare expenditures in proportional prize contests between participants who receive anodal stimulation over rTPJ and those who receive sham and cathodal stimulation. In the task, there are 2 participants in a group. They have to expend irreversible resources to compete for their share of the prize, which is equal to the ratio of the individual's expenditure to the aggregated expenditure. The individual's total earning in the contest is a participant's endowment minus the expenditure and plus her/his share of the prize. Therefore, bidding too aggressively could cause unnecessary cost even if winning some share of the prize. Participants have the motivation to bid more rationally. If a participant in the contest could make a more precise inference about the other's strategy, he or she could formulate more correct belief about the opponent, and then follow a strategy more closely to rational bidding. Overbidding will thus be reduced.

While performing in the contest, participants received 1.0 mA active anodal, cathodal, or sham tDCS, in a between-subject design. When receiving anodal stimulation the activation of rTPJ is enhanced the activation of rTPJ is enhanced and the participants' performance or accuracy of inferring or adapting the others' intentions will be improved, which in turn will allow the participant to bid more rationally. If so, we expect that anodal tDCS will lead to reduced overbidding. We also expect that a participant's expenditure will be decreased in the anodal condition compared with the sham condition. To the best of our knowledge, this study is one of the first to use tDCS to explore the function of rTPJ in processing strategic choice in competitions. We test the hypothesis that rTPJ is causally involved in overbidding based on the findings of Van den Bos et al. (2013). The results may enable us to investigate the causal relationship between the neural function of inferring others' mental states and overbidding in a contest.

## Materials and Methods

### Participants

A total of 92 healthy participants from Nankai University were recruited to participate in our experiment. All participants were right-handed without ex ante knowledge of tDCS or the contest. There was no history of psychiatric illnesses or neurological disorders for any of the participants. The data from 2 participants in the sham condition were excluded from this study because they did not understand the instrument. Lastly, 90 participants (35 males and 55 females; mean age: 21.711, ranging from 18 to 35 years) were retained in the sample. The participants were randomly assigned to receive anodal tDCS (*n* = 30, men: 11; mean age: 21.967, SD = 1.691), cathodal stimulation (*n* = 30, men: 9; mean age: 21.100, SD = 1.729), and sham stimulation (*n* = 30, men: 15, mean age: 22.067, SD = 3.258). Written informed consent was obtained from each participant prior to the study. The experiment was performed in accordance with the Declaration of Helsinki and was approved by the ethics committee of Nankai University. Each participant was paid based on their own decisions. On average, each participant received 55 Chinese Yuan (~US $8.30). None of the participants reported any adverse side effects concerning pain on the scalp or headaches after the experiment.

### Transcranial Direct Current Stimulation

tDCS is a non-invasive technique by which a weak electrical current is applied at the scalp using two electrodes placed on target cortical regions (DaSilva et al., 2011) via two saline-soaked surface sponge electrodes (35 cm^2^). These electrodes are typically referred to as the anode and cathode and are attached to separate locations. Currents between 0.5 and 2 mA intensity are applied by positioning electrodes on the brain regions of interest with polarity stimulation. As the flow emanates from the single anode electrode and then returns by way of the cathodal electrode, the membrane is depolarized and the neuronal firing rates are improved (Nitsche and Paulus, 2000). Thus, anodal tDCS enhances and cathodal tDCS diminishes cortical excitability in the short term (<5 min) and long term (up to 120 min, after at least 7 min stimulation) (Jacobson et al., 2012). tDCS-induced after-effects on social cognition and decision making have been widely observed (Pirulli et al., 2014; Sellaro et al., 2016; Jamil and Nitsche, 2017).

**Figure 1:**
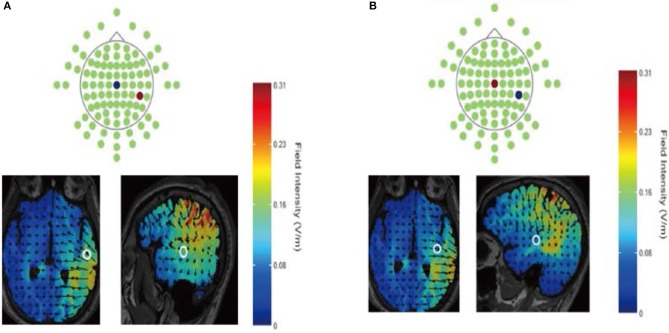
Direct current flow during anodal **(A)** or cathodal **(B)** stimulation using the HD-Explore software (Soterix Medical Inc., New York). Electrodes were placed over the CP6 and Cz according to the International EEG 10–10 system. The anode is depicted in red; the cathode is depicted in blue.

In our experiment, the current was constant and delivered by a battery-driven stimulator (Neuro Conn, Germany). The anodal or cathodal electrode was placed over the CP6 according to the International EEG 10-10 system, and the reference electrode was secured over the vertex. The current was constant at 1 mA intensity with 15 s of ramp up and down. The direct current flow and the location of the electrodes are shown in Figure 1. Computer simulation confirmed that the CP6 and Cz montages targeted the rTPJ and vertex respectively and the current flow pattern could be validated. For the sham stimulation, procedures were the same but the current lasted only for the first 30 s. The participants might have felt the initial itching even though there was no current for the rest of the stimulation.

We chose this design based on our review of previous tDCS studies. First, the CP6 was chosen frequently for non-invasion stimulation targeted at the rTPJ (Donaldson et al., 2015). tDCS studies of imitation inhibition or social ability (Hogeveen et al., 2015; Santiesteban et al., 2015) and moral judgment (Sellaro et al., 2015; Tang et al., 2017) located the rTPJ at the CP6. All aforementioned preceding studies were focused on social cognition, which is similar to the current research. Second, some studies suggested high intensities did not necessarily yield substantial after-effects. An intensity of 1 mA could produce an equivalent anodal effect relative to other intensities, while uniquely resulting in sustained excitability diminution in cathodal stimulation (Pirulli et al., 2013). Meanwhile, our stimulation duration time and intensity remained consistent with previous tDCS studies targeting the rTPJ (Santiesteban et al., 2015). Finally, offline (rather than online) stimulation was chosen because several previous studies have shown that the effects might be more robust for the former than the latter simulation (Pirulli et al., 2013). There was also another study confirming that offline stimulation led to a similar impact on cognitive tasks (Axelrod et al., 2015).

### Task and Procedure

After 20 m of stimulation, participants were asked to complete the main task, that is, the proportional prize contest. The task used by previous studies to link the rTPJ to overbidding was a common value auction (Van den Bos et al., 2013). There was also a neuroimaging study that employed a first price private value auction and indicated the striatum as a part of the neural network underlying overbidding (Delgado et al., 2008). In the auction, the highest bid wins the prize with certainty, while the other losing bidders need not to pay their bids. However, most competitions have the same characteristics of a contest, in which greater expenditure could only improve the probability or share of the prize won. The contestants will always pay their expenditures no matter if they win or lose. Thus, subjects are more motivated to compete in contests compared to auctions and may exert more effort to infer others' strategies to win the game via optimal cost. There are several types of contests and one of the differences between them is how the prize is distributed. In a winner-take-all lottery contest model (Tullock, 1980; Sheremeta, 2013), the winners take the entire prize, while the losers get nothing. In a proportional prize contest, the prize is distributed according to each player's bidding. Behavioral studies have confirmed that proportional prize contest would reduce overbidding and its sensitivity to noise in performance function (Chowdhury et al., 2014) compared to winner-take-all contest. Thus, we employed a proportional prize contest (Cason et al., 2010) as our main experimental task.

Our experiment involved *N* = 2 participants in a group, in which one of the two participants was indexed by *i* and the other one was indexed by *j*, and the participants received their endowment of *E* = 80francs at the beginning of every period. The size of the prize *V* was 80 francs. The participants were allowed to invest any amount between 0 and 80 in the contest to receive their share of the prize. Let *e*_*i*_ be the participant*i*'s expenditure and *e*_*j*_ be the participant*j*'s expenditure; the proportion of the prize participant *i* could share was: $P_{i}=\frac{e_{i}}{e_{i}+e_{j}}$, given *e*_*i*_ + *e*_*j*_ > 0. If *e*_*i*_ = 0 and *e*_*j*_ = 0, then each one will receive the prize with a probability of 0.5. Participant*i*'s total earning in every period was their endowment minus their expenditures, plus their share of the prize. Thus, Participant*i*'s total earning in every period was *E* − *e*_*i*_ + *P*_*i*_ × *V*. It has been proved that a unique pure Nash equilibrium of the contest exists (Szidarovszky and Okuguchi, 1997) participant's goal was to maximize the expected utility function as follows:

(1) $$\begin{array}{l} \text{Max }E-e_{i}+P_{i}\times V \end{array}$$

Differentiating above function with respect to *e*_*i*_, we get:

(2) $$\begin{array}{l} -1+\frac{e_{j}}{{\left(e_{i}+e_{j}\right)}^{2}}V=0 \end{array}$$

Which is equivalent to $e_{j}V={\left(e_{i}+e_{j}\right)}^{2}$. The solution of this equation leads to $\sqrt{e_{j}V}-e_{j}=e_{i}$ which is *i*'s best response, given *j*'s expenditure. Asymmetrically, we could get *j*'s best response $\sqrt{e_{i}V}-e_{i}=e_{j}$. We thus have $\sqrt{e_{j}V}-e_{j}=\sqrt{e_{i}V}-e_{j}$, that is, on the optimal level of investment in contest, we should have *e*_*i*_ = *e*_*j*_. From this observation, it follows that $e_{j}V={\left(e_{i}+e_{j}\right)}^{2}$ could be replaced with $e_{j}V={\left(2e_{j}\right)}^{2}$, and then we get the Nash equilibrium $e_{i}=e_{j}=e^{\text{*}}\text{=}\frac{V}{4}$. The Nash equilibrium expenditure is the optimal size a participant could expend in the contest, and any expenditure above it is strictly dominated (Tullock, 1980). In the current study, since *V* = 80 we have e^*^ = 20, which means that the individual Nash equilibrium expenditure is 20. It is also the rational and optimal expenditure predicted by standard game theory (Szidarovszky and Okuguchi, 1997; Sheremeta, 2013).

The experiment was processed for 20 periods. In each period, participants simultaneously input their expenditures for the contest without knowing what their opponents' bid was in this period. After the expenditures were decided, a computer calculated the outcomes, and showed participants their share of the prize, the size of the prize they won, and their total earning in this period. The participants were re-matched randomly in each period. They were told that there were sufficient opponents who would play with them online. In fact, it was the computer working as opponents to generate their bids for the 1st−20th period, which were 19, 22, 25, 27, 30, 26, 29, 31, 30, 27, 29, 31, 34, 37, 39, 39, 25, 26, 34, and 37. Thus, the bids were presented to every participant with the same pseudorandomized order. After the participants completed all the experiments, 2 periods were randomly selected for payment. After the experiment, the participants reported their demographic characteristics including gender, major, grade point average (GPA), age and per capita family monthly income.

### Data Analysis

We used the participants' expenditures in every period as the proxy of bidding behavior in the contest and compared these expenditures across three stimulation conditions to determine whether a change of activation in the rTPJ affected overbidding. To measure overbidding, we denoted the overbidding rate as the extent to which the average expenditure was over the Nash equilibrium. In each period of each condition, we summed all 30 participants' expenditures and calculated the mean. Let ${e^{\prime }}_{jt}$ be the average expenditures in the *t*th period of condition *j* (*j* = anodal, cathodal and sham condition), then the overbidding rate in each period of each condition was defined as:$\frac{{e^{\prime }}_{jt}-e^{\text{*}}}{e^{\text{*}}}$. It is also the rational and optimal expenditure predicted by standard game theory (Szidarovszky and Okuguchi, 1997; Sheremeta, 2013). If the overbidding rate is greater than zero, then we observe overbidding. The larger this proxy is, the more significant the overbidding becomes.

Thereafter, the overbidding rate and individual expenditures were separately entered in a one-way ANOVA with stimulation (anodal, cathodal and sham), followed by Bonferroni adjusted *post-hoc* tests for paired comparison. When data did not meet the homogeneity variance assumption, we applied a non-parameter test. We then applied *t*-tests to compare demographic data across the three stimulation conditions. To additionally examine the causal effect of tDCS on the rTPJ and overbidding while controlling demographic characters, we applied a panel random effect regression with standard errors clustered at the individual level. We calculated individual overbidding, which was the individual expenditures minus the Nash equilibrium (which was 20) in every period, and used it as the dependent variable. Anodal (anodal = 1, others = 0) and cathodal (cathodal = 1, others = 0) were the dummy variables that were used to estimate the tDCS effect. Control variables included demographic variables. Female (female = 1, male = 0) and major (Economics and Management = 1, other majors = 0) were binary variables. GPA was the participant's GPA score. Income was the logarithm of a participant's per capita family monthly income.

## Results

**Figure 2:**
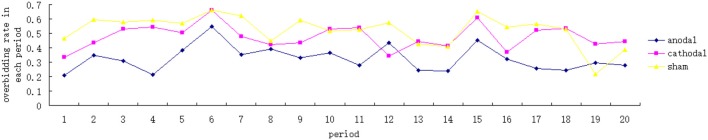
Overbidding rate in each period across the three conditions.

**Figure 3:**
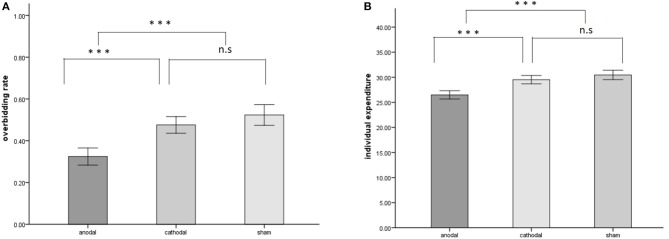
Stimulation effects (anodal/cathodal vs. sham) on the overbidding rate **(A)** and individual expenditures **(B)** across all periods. A significant main effect of both overbidding rate and individual expenditures was observed, driven by the difference between the anodal condition and other conditions. Error bars depict SD of the mean, ***p < 0.001.

The overbidding rate in the 20 periods is plotted in Figure 2, the mean and standard error of overbidding rate in alternative stimulation conditions are depicted in Figure 3A. Overbidding was observed in all three conditions. The average expenditures in every period exceeded the Nash equilibrium. The difference between the expenditure and Nash equilibrium was significant for all conditions [for anodal, *t*_(599)_ = 15.263, *p* = 0.000; for cathodal, *t*_(599)_ = 22.312, *p* < 0.001; for sham, *t*_(599)_ = 21.956, *p* < 0.001]. The overbidding rate in every period ranged from 0.21 to 0.55 in the anodal condition, from 0.33 to 0.66 in the cathodal condition, and from 0.22 to 0.66 in the sham condition (see Table 1 for the summarized results). A one-way ANOVA of the overbidding rate indicated that overbidding was significantly influenced by stimulation, thereby suggesting the main effect of the conditions [*F*_(2,57)_ = 24.749, *p* < 0.001]. *Post-hoc* tests (Bonferroni) showed a significant difference between the sham and anodal conditions (*p* < 0.001) and between the cathodal and anodal conditions (*p* < 0.001). By contrast, the difference between the sham and cathodal conditions was not significant (*p* > 0.1). The overbidding rate was significantly higher in the sham condition than in the anodal condition [for anodal, *t*_(38)_ = −6.469, *p* < 0.001] but not different from the cathodal condition [*t*_(38)_ = −1.568, *p* = 0.125]. The difference between the anodal and cathodal conditions was significant (*t*_(38)_ = −5.533, *p* < 0.001], thereby suggesting that the overbidding in the anodal condition was lower than that in the cathodal condition.

**Table 1:** Mean (SD) Overbidding rates, as a function of stimulation condition, *N* = 20 periods.

	***N***	**Min**	**Max**	**Mean**	**SD**
Anodal	20	0.21	0.55	0.325	0.088
Cathodal	20	0.33	0.66	0.476	0.085
Sham	20	0.22	0.66	0.523	0.106

Table 2 summarizes the individual expenditures, in which N is the population of participants in each condition multiplied by number of periods. The average expenditures in the anodal, cathodal, and sham groups were 26.490, 29.515, and 30.467, respectively. A one-way ANOVA of the participants' expenditures indicated that bids were significantly influenced by stimulation, thereby suggesting the main effect of the conditions [*F*_(2,1797)_ = 21.927, *p* < 0.001). *Post-hoc* tests (Bonferroni) showed a significant difference between the sham and anodal conditions (*p* < 0.001) and between the cathodal and anodal conditions (*p* < 0.001). Meanwhile, the difference between the sham and cathodal conditions was not significant (*p* = 0.388). However, the data from expenditures did not meet the homogeneity of variance, which is the standard assumption of ANOVA. Thus, we used a Kruskal-Wallis test as a robust check. The non-parameter test results also confirmed a significant main effect, χ^2^(2) = 55.309, *p* < 0.001. Thereafter, we applied the Mann-Whitney U test to compare the bid among the three conditions: sham, anodal, and cathodal. The results revealed a significant difference between the anodal and sham conditions (*z* = −6.802, *p* < 0.001). The difference between the cathodal and sham conditions was not significant (*z* = −1.333, *p* = 0.182). The expenditures in the anodal and cathodal conditions were significantly different (*z* = −5.922, *p* < 0.001).

**Table 2:** Summary of the individual expenditures.

	***N***	**Min**	**Max**	**Mean**	**SD**
Anodal	600	1.000	80.000	26.490	10.416
Cathodal	600	0.000	80.000	29.515	10.446
Sham	600	1.000	75.000	30.467	11.677

**Figure 4:**
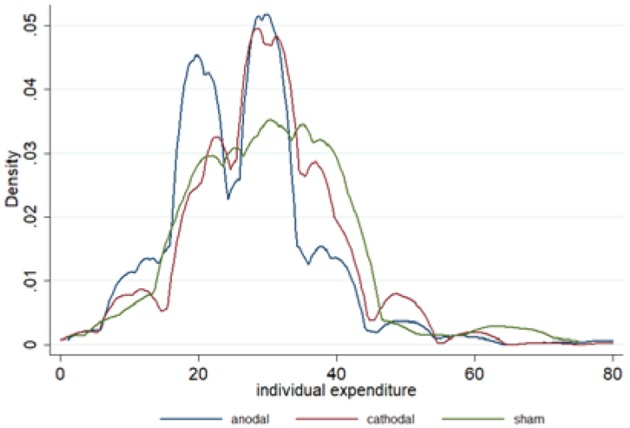
Kernel density comparison of the distribution of individual expenditures in the anodal, cathodal, and sham conditions across 20 periods. Kernel density estimates were computed using STATA 11.0 and Silverman bandwidth selection.

The mean and standard error of expenditures are depicted in Figure 3B. The kernel-smoothed distribution of the individual expenditures in each condition is plotted in Figure 4. Two peaks of the distribution of expenditures were observed in the anodal conditions. One peak nearly fit the Nash equilibrium and the other was located above the Nash equilibrium. However, the distribution in the cathodal and sham conditions was considerably smooth and its unique peak was located above the Nash equilibrium. These features demonstrate that the expenditures in the anodal condition were relatively closer to the Nash equilibrium level than those in the cathodal or sham conditions. In the sham condition, the proportion of Nash equilibrium biddings was 15.67%, which was significantly lower than that in the anodal condition (20.67%, Fisher's Exact test, *p* < 0.001), but not different from the cathodal condition (12.33%, Fisher's Exact test, *p* = 0.114). The amount of biddings above the Nash equilibrium was less in the anodal condition compared with the sham condition. In the sham condition, the proportion of expenditures above the Nash equilibrium was 73.83%, which was significantly greater than that in the anodal condition (62%, Fisher's Exact test, *p* < 0.001), but not different from the cathodal condition (77.5%, Fisher's Exact test, *p* = 0.158). Meanwhile, the proportion of the expenditures above the Nash equilibrium in the anodal condition was significantly decreased compared to those in the cathodal condition (*p* < 0.001).

We ran panel regression with random effect to further assess the effects of the anodal and cathodal stimulations on the participants' overbidding behavior in contests. Table 3 shows the results. It was anodal (*coeffi*. = −3.644, *p* = 0.033), rather than cathodal (*coeffi*. = −0.615, *p* = 0.825) stimulation that significantly reduced overbidding in the contest. In addition, the effect of tDCS was unchanged when we controlled for demographic variables. These regression results further provide robust evidence that is consistent with the mean comparison results. In the regression we controlled the demographic characteristics, such as gender, major, GPA, age, and per capita family monthly income. Table 3 reports the coefficients and significance. Evidently, the effect of tDCS was unchanged when we controlled for the aforementioned demographic variables.

**Table 3:** Effect of tDCS on overbidding.

**Dependent variable: overbidding**	**Total**	**Anodal**	**Cathodal**	**Sham**
Anodal	−3.644^**^ (1.70)			
Cathodal	−0.615 (1.77)			
Female	−0.691 (1.64)	2.866 (1.98)	0.321 (2.88)	−4.400 (2.94)
Age	0.183 (0.23)	0.793^*^ (0.46)	0.302 (0.78)	−0.193 (0.27)
Major	2.241 (1.36)	0.600 (1.86)	2.502 (2.07)	3.928 (2.53)
GPA	−0.663 (0.84)	−1.220 (1.00)	−2.092 (2.12)	1.240 (1.67)
Income	−0.391 (0.79)	−0.0755 (0.93)	0.916 (1.57)	−2.215 (1.69)
Constant	30.35^***^ (9.63)	10.60 (12.86)	18.89^*^ (10.68)	50.48^***^ (18.48)
*N*	1780	580	600	600

Panel estimation on tDCS effect. Dependent variable: individual expenditures minus Nash equilibrium (which was 20) in every period. Robust standard errors in parentheses.

*** *Indicates significance at the 1% level*,

** *at 5%*,

* *at 10%. All models include participant individual random effect error structure. Standard errors are clustered at the individual level*.

Finally, we compared the demographic characteristics, such as gender, major, GPA, age, and per capita family monthly income across the conditions. There was no significant difference in the population of female participants between the anodal and sham conditions [Fisher's exact test, *p* (2-sided) = 0.435], or the cathodal and sham condition [Fisher's exact test, *p* (2-sided) = 0.187]. This situation is also true for the comparison of ratios of major in the anodal/cathodal and sham conditions [Fisher's exact test, *p* (2-sided) = 0.301/0.796]. Participants' average age in the anodal condition was marginally higher than that in cathodal condition [*t*_(58)_ = 1.963, *p* = 0.055]. No other significant differences in age, GPA, or per capita family monthly income were observed across the three conditions (all *p*-values > 0.1).

## Discussion

In the present study, we aim to elucidate the neural mechanism underlying overbidding, and highlight the motivation of the expenditures in contests through evidence from a neuroscientific approach. tDCS was employed to investigate the causal relationship between activation of the rTPJ and overbidding in proportional prize contests. Specifically, we tested whether 1.0 mA low current electrical stimulation could influence participants' expenditures in contest and, thus, impact overbidding. We then compared the overbidding rate and participants' expenditures in the anodal and cathodal conditions with those in the sham condition. The results showed that the rTPJ plays an important role in contests. Overbidding was significantly reduced after participants received anodal stimulation. However, compared with the sham condition, the effect of cathodal stimulation was not significant. A panel data regression analysis with standard error clustered at the individual level was run to further examine whether stimulation or demographic characteristics could predict overbidding. We confirmed that only anodal stimulation could significantly impact the overbidding trend, and cathodal stimulation did not have a significant effect. These results could not be explained by the individual heterogeneity of the demographic characteristics. Thus, our experimental results provide direct evidence for the argument that the rTPJ is an important neural basis of overbidding.

Overbidding means that the average effort exerted in competition is significantly higher than the standard Nash equilibrium prediction. This phenomenon involves cognitive processes such as inferring others' intention. Overbidding occurs often in contest in which the contestant's total earning equals to the endowment, minus the irreversible expenditure, plus the prize they wins. Greater expenditure could improve the probability or share of the prize, but may also reduce the remaining endowment. Whether the gain is increased or decreased depends on each other's expenditure. Thus, participants are motivated to infer others' strategies more precisely to win the game via optimal expenditure. Only when participants infer that other opponents will choose the same normative choice and trust that decision could the individual choices converge to make the optimal bid (Sheremeta, 2013; Fehr and Schurtenberger, 2018). Several recent experimental studies have indicated that the accuracy of inferring others' strategy might be relevant to irrational expenditure and overbidding in contest and determined a positive correlation between participants' expenditures and belief (Rockenbach and Waligora, 2016; Sheremeta, 2016). If it is the case, the more correct belief the participants may hold, the more rationally they may bid. In addition, if at the beginning players found their predictions were different to what other players actually did, they would try to change their strategies based on inference of others' intentions or strategies. Taken together, if a participant in a contest could make a more precise inference about others' strategies, and bid more rationally, he or she may obtain greater earnings via optimal expenditure. Overbidding will decrease with a population of these more rational contestants. Therefore, the accuracy of predicting others' strategies, or mentalizing related strategic computation, will be the crucial neural mechanism which impacts overbidding.

A possible explanation of our experimental results might be that activity change in the rTPJ induced by anodal stimulation could improve participants' accuracy in mental state inference and decrease overbidding via a change in participants' bidding behavior. This speculation was consistent with the arguments that the rTPJ was associated with the inference of intention or choice underlying others' social behavior (Saxe and Kanwisher, 2003; Saxe and Wexler, 2005; Krueger et al., 2008). In particular, the activation in rTPJ was engaged into competitive games with substantially strategic uncertainty (Halko et al., 2009; Polosan et al., 2011; Votinov et al., 2015). rTPJ activity reflects the demand of perspective taking and strategic thinking in competitions, i.e., when participants try to infer other participants' mental states or when participants try to be unpredictable (Tsoi et al., 2016) or avoid loss (Votinov et al., 2015). rTPJ is also involved in the neural computation process when people need to predict others' strategies and estimate the influence of their actions on opponents' actions (Hampton et al., 2008; Bhatt et al., 2010; Volz et al., 2015; Hill et al., 2017). The more through a participant was in trying to reason their opponents' choices, the more activation in rTPJ was found. In addition, rTPJ response patterns may reflect predictive errors encoding and resulting belief adaption process in competition (Bitsch et al., 2018). tDCS studies also provided evidence that rTPJ could play a critical role in inferring others' intention in moral judgement (Ye et al., 2015; Mai et al., 2016) or sensitivity of others' pain (Coll et al., 2017). Regarding our results, there was a peak that nearly fit the Nash equilibrium on the kernel density curves only in the anodal condition, which could be interpreted as there being more Nash equilibrium bids in the anodal condition compared to the other two conditions. In contrast, the amount of biddings above the Nash equilibrium was significantly less in the anodal condition compared with the cathodal and sham condition. Meanwhile, the expenditures in the sham condition were obviously greater than those in the anodal condition, and the overbidding rate in the sham condition was also significantly greater. These results suggest that anodal stimulation enabled participants to make better inference about others' strategies through enhancing activation of rTPJ, and then improved participants' accuracy in strategic decision making. Therefore, the participants in anodal condition bid more rationally, which in turn reduced overbidding.

In addition, a different line of studies provide evidence in favor of the argument that rTPJ contributes to altruism and generosity. For instance, Morishima et al. (2012) demonstrated that rTPJ played a special role in altruistic behavior when participants made decisions in advantage inequality domain. Morishima et al. (2012) also found rTPJ was strongly activated when the cost of altruistic behavior was close to the maximal cost a participant was willing to bear, thus indicating a link between the pattern of functional activity in rTPJ and participant-specific altruistic tendency. Strombach et al. (2015) confirmed rTPJ was associated with generous decisions, and they further found rTPJ was important for overriding selfish impulse. In a recent study, Tang et al. (2017) revealed that enhanced activation of rTPJ reduced deception and promoted fair behavior in a dictator game in which participants could lie about the stake needed to be allocated. Luo et al. (2017) also found stimulating rTPJ lead to fairer distribution and more aversion to inequality. Taken together, rTPJ may inhibit selfish motivations and be associated with pro social behavior. Therefore, another explanation of our results may be that enhanced activity in rTPJ improved participants' concern with their opponents' well-being, thus reducing expenditures and overbidding.

We observed an effect of anodal rather than cathodal stimulation. Substantial research on cognitive tasks could determine the anodal excitation effects, whereas limited studies could obtain the cathodal effect or anodal excitation/cathodal inhibition effect (Jacobson et al., 2012). The lack of a cathodal inhibition effect may be the result of the cognitive functions being supported by a considerably extensive neural network rather than motor function (Jacobson et al., 2012). Brückner and Kammer (2017) suggested that cathodal stimulation having an ineffective, inhibitory, or improved effect (e.g., Weiss and Lavidor, 2012) might depend on many factors, such as the neural networks, participants' experience (Miniussi et al., 2013), or timing of stimulation (Brückner and Kammer, 2017). More recently, other stimulation works found that the effect of cathodal stimulation may be dependent on the initial cognitive activation level (Benwell et al., 2015). One possible interpretation of the absence of a cathodal effect is that the baseline effort participants exerted to infer others' strategy were already at a low level, thus cathodal stimulation could not generate enough inhibition to increase overbidding. There is evidence for this speculation. The proportion of expenditures above the Nash equilibrium in the sham and cathodal conditions was greater than that in the anodal condition, which indicated participants' performance on mental state inference was relatively lower in the sham condition. Indeed, some studies found that while anodal stimulation changed behavior in some social cognition tasks, the cathodal inhibition effect was not observed (Santiesteban et al., 2012; Sellaro et al., 2015). Future work may still need to address this issue.

It should be acknowledged that there are some disadvantages that limit the conclusions of our experiments. Firstly, as we discussed above, the simulation effect may be caused by improved accuracy of inferring others' mental state, or more concerns with the opponents' well-being. The present results couldn't distinguish the two explanations from each other or reveal their comprehensive effect on overbidding. Future works may need to study the role of rTPJ in overbidding with more details and clarify the mechanisms. Secondly, in the present study we applied tDCS at 1 mA. Our simulation confirmed that this intensity could lead to an efficient stimulation targeting the rTPJ. Several studies applied higher (1.5–2 mA) intensity stimulation to achieve significant after stimulation results in social cognition tasks. For instance, some studies applied tDCS of 1.5 mA (Mai et al., 2016) or 2 mA (Coll et al., 2017) over rTPJ to provide a link between activation of the rTPJ and intention inferring (Mai et al., 2016). However, there are also studies using 0.5 mA current intensity as a control condition in a working memory task (Brunyé et al., 2017). Some previous evidence has validated our choice of the current intensity (Santiesteban et al., 2012, 2015; Hogeveen et al., 2015; Jamil et al., 2016). Our data also showed a significant anodal excitability effect, which provided some support for our design. Continuing research could use a 1.5–2 mA intensity to additionally test the robustness of the causal relationship between stimulation and behavioral changes. Third, some works have suggested that other regions may be related to overbidding, such as the vmPFC or rIFG. Furthermore, the rTPJ-vmPFC connectivity strength after winning was also correlated with subjective value in donation decision making (Hare et al., 2010). Since some studies have shown that the vmPFC was correlated with subjective value in donation decision making (Hare et al., 2010) and tracked the motivational value of rewards (Azzi et al., 2012), the correlation between the rTPJ-vmPFC connectivity and overbidding may reflect an interaction of the rTPJ and value computation systems during social context (Hare et al., 2010), especially in competition. The rTPJ may influence the degree to which the vmPFC activates and then modulates subjective motivational value of competition results. Thus, future works are still required to investigate the role of this connectivity in overbidding by stimulating the rTPJ.

## Conclusions

This study demonstrates that manipulation of activity in the rTPJ has a causal effect on overbidding and bidding behavior, probably through the improved performance or accuracy of inferring others' choices or strategies. By using a proportional prize contest while delivering tDCS on the rTPJ, we found that the anodal tDCS reduced overbidding in a contest compared with the cathodal and sham stimulations and improved the proportion of Nash equilibrium biddings. These results were robust even when we controlled demographic characteristics. Future work could study the role of rTPJ in more detail to test whether this anodal stimulation effect could contribute to increased concern for others, investigate the consequences of higher intensity current on the rTPJ when participants are competing in contests, or investigate the role of the rTPJ-vmPFC connectivity in overbidding.

## Ethics Statement

A written informed consent was obtained from each participant prior to the study. The experiment was performed in accordance with the Declaration of Helsinki and was approved by the ethics committee of Nankai University.

## Author Contributions

MW, JL, and DL designed the experiment. MW, JL, and CZ performed experiment. MW, JL, and CZ analyzed the data. MW and CZ drew figures. MW and JL wrote the manuscript.

### Conflict of Interest Statement

The authors declare that the research was conducted in the absence of any commercial or financial relationships that could be construed as a potential conflict of interest.
